# Can public-domain datasets be leveraged to identify factors associated with the occurrence of African swine fever in europe?

**DOI:** 10.1186/s13028-025-00832-7

**Published:** 2025-11-20

**Authors:** Ofosuhene Okofrobour Apenteng, Ana Rita Pinheiro Marques, Lene Jung Kjær, Beate Conrady

**Affiliations:** Department of Veterinary and Animal Sciences, Section for Animal Health and Welfare, University of Copenhagen, Copenhagen, Denmark

**Keywords:** ASF, EMPRES-i, Pigs, Wild boar, WOAH-WAHIS

## Abstract

**Background:**

African swine fever (ASF) is a highly contagious and deadly viral disease affecting domestic pigs and wild boars. This study uses public domain datasets to identify the association between (a)biotic variables and occurrences of ASF in domestic pigs and wild boars in Europe. The public domain databases WOAH-WAHIS and EMPRES-i were used to obtain data about ASF cases in domestic pigs and wild boars from 2018 to 2023. Several (a)biotic variables were considered as potential drivers for ASF: precipitation, temperature, human-animal interface density, and type of land cover. A Shiny app was created to offer an interactive platform for data analysis and visualisation.

**Results:**

Uni- and multivariable mixed negative-binomial models were used to assess the association between (a)biotic variables and ASF occurrence. The statistically significant associations between the (a)biotic variable ‘land cover’ and ASF cases differ between domestic pigs and wild boars. The land cover types ‘industrial, commercial, and transport units’ and ‘inland wetlands’ were identified as significant factors associated with ASF in both domestic pigs and wild boars. However, pig density, temperature, and human density were statistically associated with ASF occurrence only in domestic pigs. When a finer spatial resolution (5 × 5 km) was applied for all (a)biotic variables around the reported ASF cases compared to the coarser resolution of 10 × 10 km, the associations with (a)biotic variables for wild boars remained consistent. In contrast, for domestic pigs, only human population density remained significantly associated with ASF occurrence at this finer scale.

**Conclusions:**

The model showed high accuracy for ASF prediction in domestic pigs but low accuracy for wild boars, highlighting the limitations of public domain (a)biotic factors alone. Integrating restricted data on animal movements, migration, and carcass interactions could enhance future predictions and improve disease control strategies. The change in spatial resolution did not affect the associations between (a)biotic factors and ASF occurrence in wild boars but reduced the number of associated variables in domestic pigs, suggesting that ASF in wild boars is driven by broader-scale factors, while in domestic pigs it is influenced by more localised conditions.

**Supplementary Information:**

The online version contains supplementary material available at 10.1186/s13028-025-00832-7.

## Background

Domestic pig and wild boar populations can be impacted by the highly contagious and fatal virus known as the African swine fever (ASF) virus [1–4]. In 1921, the first ASF case was discovered in Africa, and in the early 1950s and 1960s, the disease spread for the first time to Europe and other regions of the World, respectively [5]. Africa reported 128 outbreaks covering 61,459 cases, while in Asia, 9,928 outbreaks totalled 115,309 cases, and in Europe, 4,271 outbreaks were notified, including 625,269 cases between 2016 and 2020 [6]. Since January 2022, ASF has affected over 728,000 pigs and 25,800 wild boars, resulting in more than 1,757,000 animal losses across five continents (Africa, America, Asia, Europe, and Oceania) [7]. Domestic pigs and wild boar can spread the ASF virus directly (e.g., via contact with other animals) and/or indirectly (e.g., through contaminated feed, farm equipment, clothes, and footwear) [3, 8, 9], but it is not a zoonosis [5, 10–12]. The World Organisation for Animal Health (WOAH) [13] and the Emergency Prevention System (EMPRES-i) [14] are two databases that receive reports on ASF and other disease outbreaks globally and inform other countries about the animal disease situation [13, 14].

In several parts of the European Union (EU), more wild boars have led to higher inhabited densities of wild boars on land scales [15–17]. Several studies suggest that the winter months have a significant role in the spread of ASF in wild boar [18, 19]. The spread of ASF during the autumn and winter seasons has been ascribed to the density of wild boar populations and land cover [20, 21]. Temperature and annual mean precipitation have been determined as key contributors to increase in the spread of ASF in the wild boar population [19]. In pig farming, the spread of ASF has been influenced by human activities [22]. For instance, it has been demonstrated through a spatially explicit Monte Carlo simulation model that human population density is a key factor contributing to the spread of ASF [23]. Thus, ASF may be managed effectively by targeting prevention measures in areas with a large human density [24]. Other studies showed that ASF is more widely distributed in high pig density areas [25–27]. Other studies showed that land cover, such as forest regions, impacted the spread of ASF [28, 29]. Temperature, precipitation, and land cover changes are important concerns for pigs and wild boars because they affect habitat, food availability, and health. Temperature influences thermal regulation and disease transmission, whereas precipitation affects vegetation and water sources. Changes in land cover, such as deforestation and agricultural expansion, disrupt habitats, increase human-wildlife interactions, and reduce resource availability. These environmental pressures influence the behaviour, population dynamics, and adaptive methods of both species [30, 31].

The objective of this study was to assess whether publicly available data on (a)biotic variables (e.g., temperature, precipitation, land cover, and the human-animal interface density) can serve as meaningful predictors for ASF occurrence in domestic pigs and wild boars in Europe.

## Materials and methods

The disease occurrence data for wild boar and domestic pig ASF outbreaks was obtained from the WOAH-WAHIS [32] and the EMPRES-i [33] databases for the period from 01/01/2018 to 31/12/2023.

The disease occurrence data were downloaded on 15/01/2024 from WOAH-WAHIS [32]. The data characterised a total of 139 diseases. The data included 63 variables, as described in (Additional file 1). Each data entry represents an outbreak, including one or more cases at a geographical location. The curated data from the WOAH database were filtered for outbreaks of ASF in 27 EU countries. Six variables from the available 63 variables in the database (Additional file 2 (WOAH)) were selected for further analysis:

- Outbreak start date.
- Reporting date.
- Species: either domestic pig or wild boar.
- The geographic location of the outbreak, which is expressed as decimal numbers with the format [latitude, longitude].
- Country name.
- Number of cases per outbreak.

Public-domain disease occurrence data were also obtained from EMPRES-i [30]. The dataset consists of 15 variables (Additional file 2). As for the WOAH-WAHIS database, we filtered and selected the same information from EMPRES-i. Both the WOAH-WAHIS and EMPRES-i datasets were combined based on the following variables: outbreak start date, country name, species, latitude, and longitude. A common outbreak was defined when both data sources reported outbreaks on the same date and location. We selected ASF occurrence data for both domestic pigs and wild boars in EU countries. A step-by-step description of the data structure, data cleaning process, and data analysis is available at the following GitHub link: https://github.com/ofosuheneapenteng/WiLiMan-ID-Deliverable-D4.2.

The following (a)biotic factors were assessed as potential drivers for the spread of ASF and obtained from various public sources: annual mean temperature in degrees Celsius (°C) [34], precipitation in millimeters (mm) [34], the density of pigs [10] and wild boar [35], as well as the human density expressed as 5 min of arc [36], i.e., a total number of inhabitants per grid-cell. These variables were spatially harmonised to a 10 × 10 km grid around the identified ASF cases to ensure consistency across variables and to minimise spatial bias in the modelling process. The densities of domestic pigs and wild boar are measured by the absolute number of animals per pixel: 4320 by 2160 pixels of 0.083333 decimal degrees resolution [10]. Land covers were obtained from the CORINE database (Coordination of Information on the Environment) [28] to characterise land cover across EU countries and included three levels of land cover details: Level 1 (characterised land cover in 5 factors), Level 2 (14 factors), and Level 3 (44 factors). For this study, we used Level 2 [37] (14 factors spanning heterogeneous agricultural areas, forest, and arable land) as a potential diversity associated with ASF occurrence (Additional file 1). To illustrate how spatial resolution may influence study outcomes, these variables were also harmonized to a finer spatial grid of 5 × 5 km around the reported ASF case locations.

Negative binomial regression models were used to explain ASF occurrence by (a)biotic factors (Additional file 1), as the count data of ASF occurrence indicated a significant overdispersion, i.e., the variance is larger than the mean. In the first step, a univariable negative binomial regression analysis was conducted to identify statistically significant associations between independent (a)biotics on the ASF occurrence as the dependent variable (Table 1). A multivariable negative binomial regression analysis was performed for all statistically significant variables identified in the univariable analysis (Table 2). The significance level in the model was set to *P* < 0.05. The model considers the country, latitude, and longitude as random variables that account for spatial variation, correct for autocorrelation, and compensate for unmeasured location-based factors. By including these random factors, the model captures the unpredictability specific to each country and their geographical location, thereby improving the accuracy and robustness of the analysis. Both the univariable and multivariable negative binomial regression analyses were performed separately for ASF outbreaks in domestic pigs and wild boars. The model parameters were estimated using Maximum Likelihood Estimation (MLE).

**Table 1:** Reported ASF cases in pigs and wild boar, 2018–2023

Country	Pigs: WOAH-WAHIS and EMPRES-i	Wild boar: WOAH-WAHIS and EMPRES-i
Belgium	0	564
Bosnia and Herzegovina	1145	6
Bulgaria	60	163
Croatia	132	5
Germany	8	860
Greece	5	1
Hungary	0	2102
Italy	17	869
Latvia	26	2517
North Macedonia	30	27
Poland	367	9969
Romania	3831	2229
Slovakia	5	27
Sweden	0	56
Total	**5626**	**19,395**

**Table 2:** Univariable negative binomial model of (a)biotic factors influencing ASF in wild boar, 10 × 10 km

Species	Fixed effects	Estimate	Std. Error	Z-value	*P*-value	Odd ratio	Lower Conf.	Upper Conf.
Number of observations = 3632, AIC = 13957.0, BIC = 13994.2, logLik= − 6972.5, deviance = 13945.0, df.resid = 3626								
Wild boar	Intercept	1.542	1.581	0.976	0.329	4.676	0.211	103.573
	Precipitation	− 0.384	0.554	− 0.693	0.489	0.681	0.230	2.017
	Random effects	Variance	Std.Dev.					
	Country (Intercept)	0.182	0.425					
	Longitude (Intercept)	0.124	0.353					
	Latitude (Intercept)	0.324	0.569					
Number of observations = 3643, AIC = 13986.9, BIC = 14024.1, logLik= − 6987.4, deviance = 13974.9, df.resid = 3637								
Wild boar	Intercept	1.279	0.724	1.77	0.077.	3.596	0.871	14.848
	Temperature	− 0.659	0.563	− 1.170	0.242	0.517	0.171	1.561
	Random effects	Variance	Std.Dev.					
	Country (Intercept)	0.207	0.455					
	Longitude (Intercept)	0.125	0.353					
	Latitude (Intercept)	0.324	0.569					
Number of observations = 3643, AIC = 13957.0, BIC = 13994.2, logLik = − 6972.5, deviance = 13975.0, df.resid = 3626								
Wild boar	Intercept	0.731	0.423	1.727	0.084.	2.078	0.906	4.765
	Wild boar density	− 0.272	0.385	− 0.705	0.481	0.762	0.358	1.621
	Random effects	Variance	Std.Dev.					
	Country (Intercept)	0.204	0.452					
	Longitude (Intercept)	0.125	0.353					
	Latitude (Intercept)	0.324	0.569					
Number of observations = 3632, AIC = 13956.2, BIC = 13993.4, logLik=− 6972.1, deviance = 13944.2, df.resid = 3626								
Wild boar	Intercept	0.516	0.163	3.167	0.001**	1.668	1.215	2.292
	Human density	− 0.052	0.046	− 1.116	0.264	0.949	0.866	1.039
	Random effects	Variance	Std.Dev.					
	Country (Intercept)	0.209	0.457					
	Longitude (Intercept)	0.124	0.352					
	Latitude (Intercept)	0.325	0.570					
Number of observations = 3632, AIC = 13951.4, BIC = 14062.9, logLik = − 6957.7, deviance = 13915.4, df.resid = 3614								
Wild boar	Intercept	0.453	0.197	2.296	0.022*	1.573	1.069	2.315
	Industrial, commercial and transport units	− 1.146	0.494	− 2.322	0.020 *	0.318	0.121	0.836
	Mine, dump and construction sites	0.1261	0.358	0.352	0.725	1.134	0.562	2.289
	Artificial, non-agricultural vegetated areas	0.183	0.977	0.187	0.851	1.201	0.177	8.160
	Arable land	-0.049	0.1424	− 0.344	0.730	0.952	0.720	1.259
	Permanent crops	0.113	0.216	0.519	0.603	1.119	0.732	1.712
	Pastures	-0.136	0.157	− 0.865	0.387	0.873	0.642	1.188
	Heterogeneous agricultural areas	− 0.136	0.152	− 0.895	0.371	0.873	0.648	1.175
	Forests	0.036	0.138	0.263	0.792	1.037	0.792	1.358
	Scrub and/or herbaceous vegetation associations	0.058	0.166	0.352	0.725	1.060	0.766	1.466
	Open spaces with little or no vegetation	0.226	0.649	0.349	0.727	1.254	0.351	4.476
	Inland wetlands	1.342	0.409	3.276	0.001**	3.825	1.714	8.537
	Inland waters	0.252	0.270	0.933	0.351	1.287	0.758	2.184
	Marine waters	0.157	0.666	0.236	0.813	1.170	0.317	4.317
	Random effects	Variance	Std.Dev.					
	Country (Intercept)	0.199	0.447					
	Longitude (Intercept)	0.123	0.350					
	Latitude (Intercept)	0.328	0.573					

Signif. codes: 0 ‘***’ 0.001 ‘**’ 0.01 ‘*’ 0.05 ‘.’ 0.1 ‘ ’ 1

Data on the ASF occurrence and (a)biotic factors were overlain on the surface of raster maps based on their geographical coordinates. Missing values for the (a)biotic factors in the raster maps were filled using the mean of the neighbour cell, and the common intersect was found by the extent of the spatial area of the raster data to ensure accurate spatial analysis of the country-level prediction. The model was assessed for overdispersion (i.e., the presence of greater variability in the data than expected under the statistical model) and collinearity (i.e., correlation among predictor variables). Collinearity was evaluated using the variance inflation factor (VIF), which indicates whether predictor variables independently contribute to explaining the dependent variable. Collinearity is considered problematic when the VIF exceeds a threshold of 10 [38]. To evaluate the effectiveness of our model, we employed a 70:30 data-splitting strategy, utilising 70% of the dataset for training and 30% for testing. This approach ensures that the model learns patterns from a significant portion of the input while maintaining a separate subset for performance evaluation. While the training set is used to fit the model, the test set provides an unbiased assessment of the model’s predictive ability. This technique helps prevent overfitting and confirms that the model generalises successfully to the test data. Additionally, cross-validation was conducted to assess the accuracy of the predictive models. Five-fold cross-validation was used, with the dataset split into five subsets. Each subset was used once for validation, while the others formed the training set, ensuring a robust performance evaluation. Further, a confusion matrix was created to evaluate the accuracy, sensitivity, specificity, and positive and negative predictive values for ASF based on the matches and mismatches between predictions and actual results (ROC analysis [39], Additional file 3). To identify potential correlations between (a)biotic independent variables, Pearson correlation coefficients were calculated (Additional files 3 and 4). The Moran’s I test was used to assess overall spatial autocorrelation across the study area. Specifically, ASF case data were linked to (a)biotic factors using a distance-based spatial weights matrix. Moran’s I evaluate whether the spatial distribution of a variable is random, clustered, or dispersed across the geographical region. The significance of the observed spatial autocorrelation was also statistically tested. Further, geographical maps were created where ASF cases were plotted with the (a)biotic factors over the study period.

A Shiny application was developed using R Shiny to provide an interactive platform for data visualisation and analysis, enabling the exploration of the data used and the analyses performed in this study.

The statistical analyses were conducted using the open-source statistical computing environment R [40]. The data analyses were performed using the packages ‘tmap’, ‘readxl’, ‘tidyverse’, ‘dplyr’, and ‘eurostat’. Further, the spatial analysis was performed using the following packages’ raster’, ‘sp’, ‘sf’, ‘terra’, ‘lmer4’, ‘caret’, and ‘spdep’ in the R version (4.3.2) [37]. A Shiny application was developed using the packages ‘shiny’, ‘leaflet’, ‘tmaptools’, and ‘shinydashboard’.

## Results

**Fig. 1:**
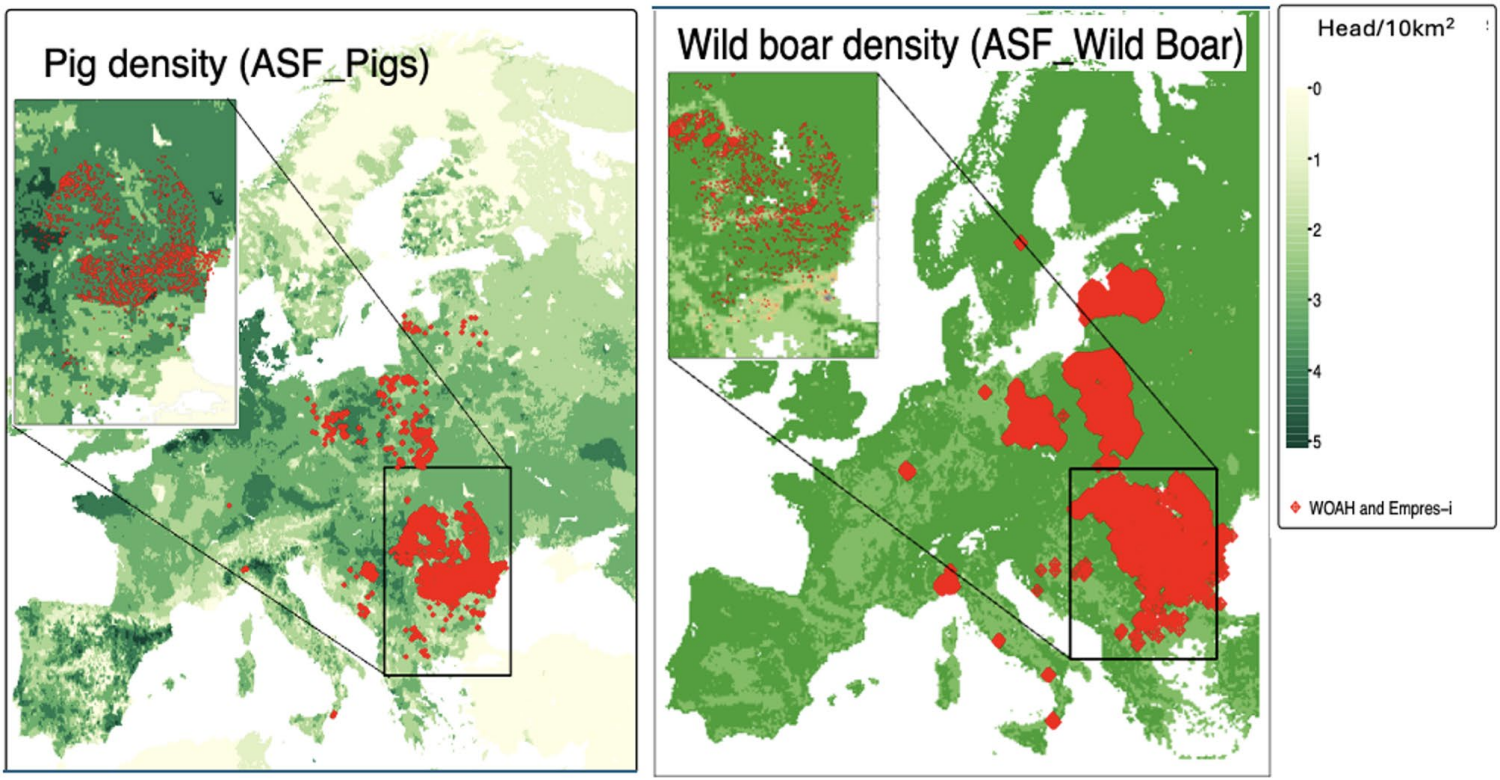
ASF cases (2018–2023) in pigs and boars overlaid on 2020 density (head/10 km²) maps

**Fig. 2:**
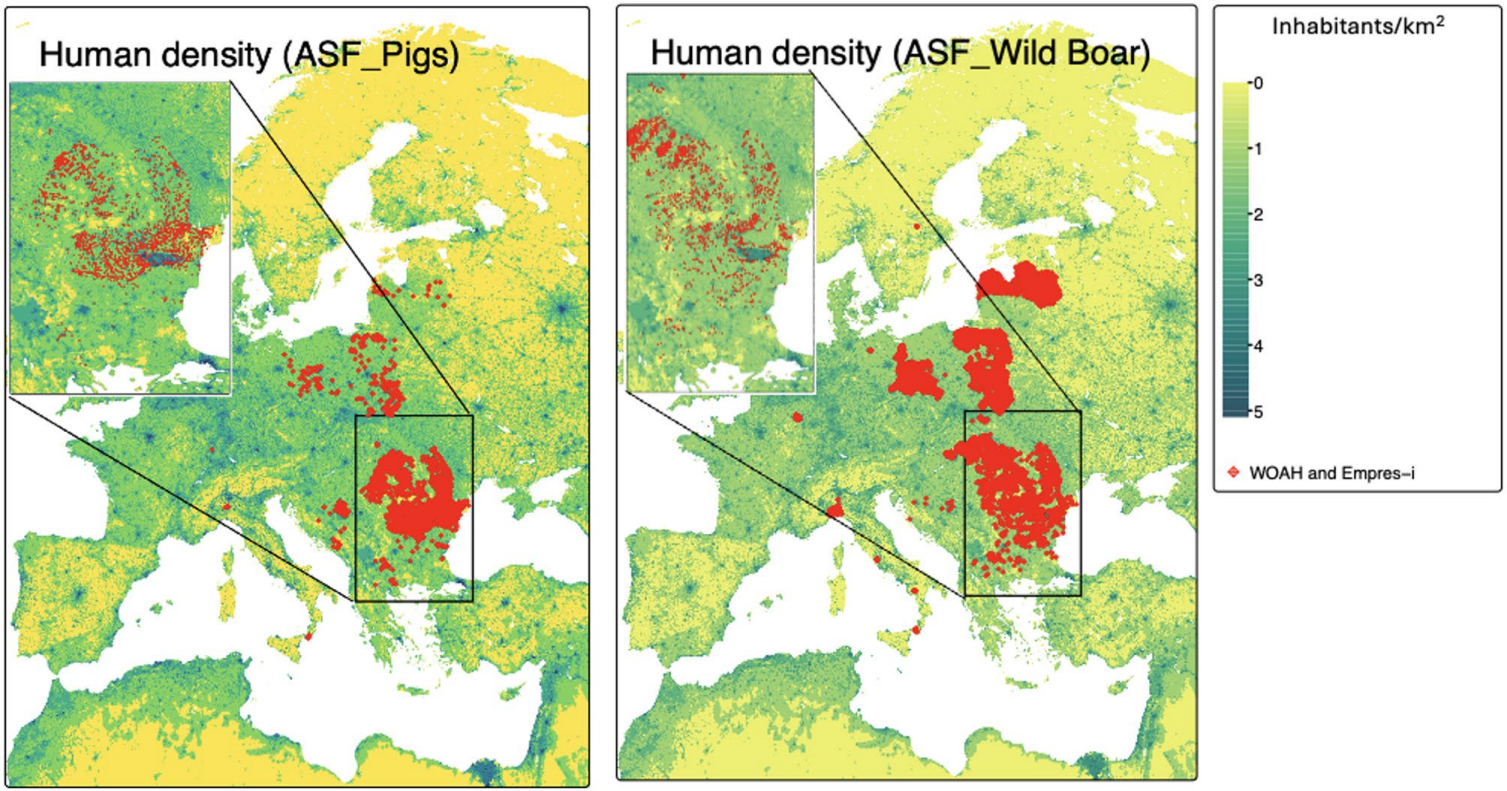
ASF cases in pigs and boars overlaid on a 2020 human density map (inhabitants/km²)

**Fig. 3:**
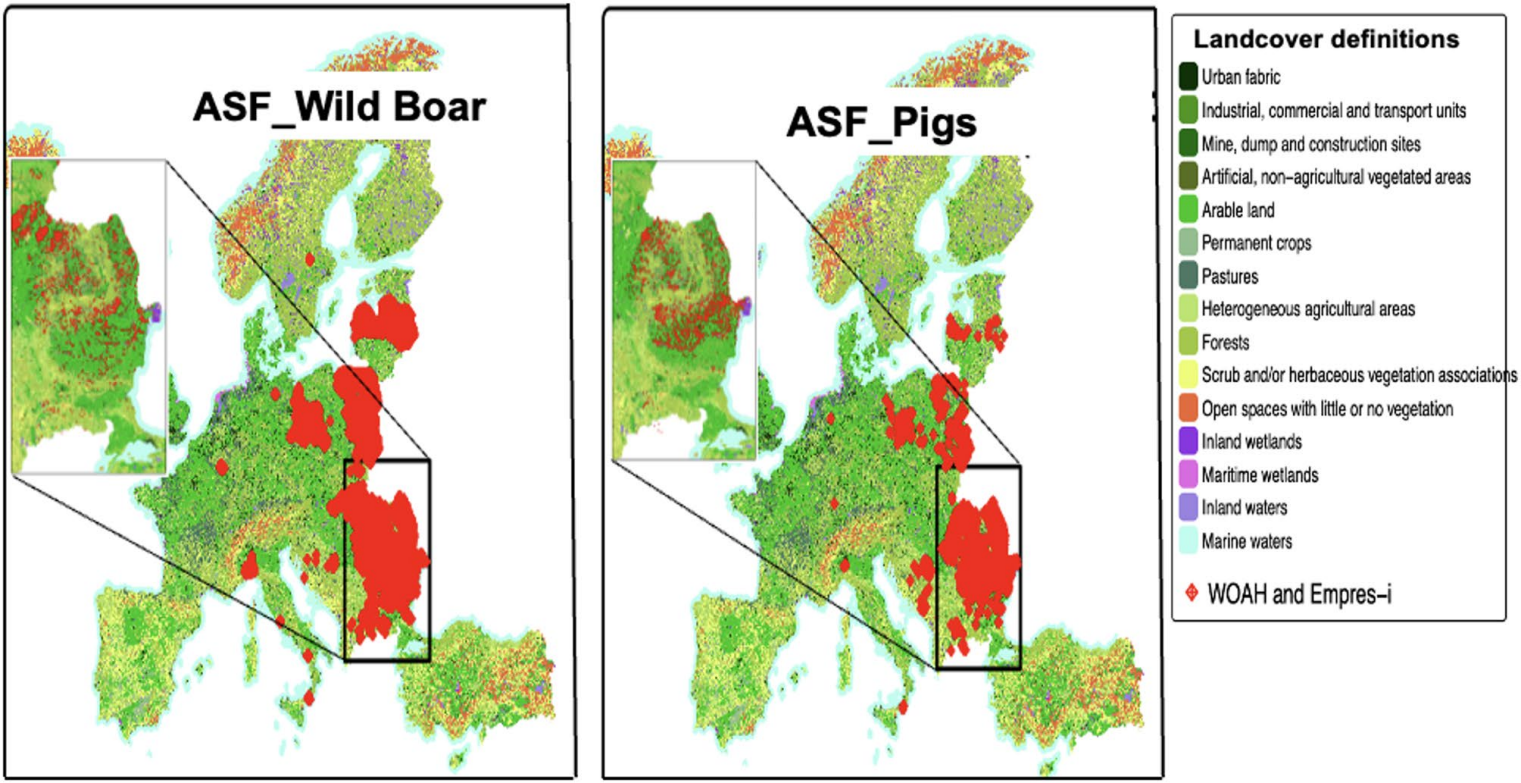
ASF cases (2018–2023) in pigs and boars overlaid on 2018 CORINE Level 2 land cover map

**Fig. 4:**
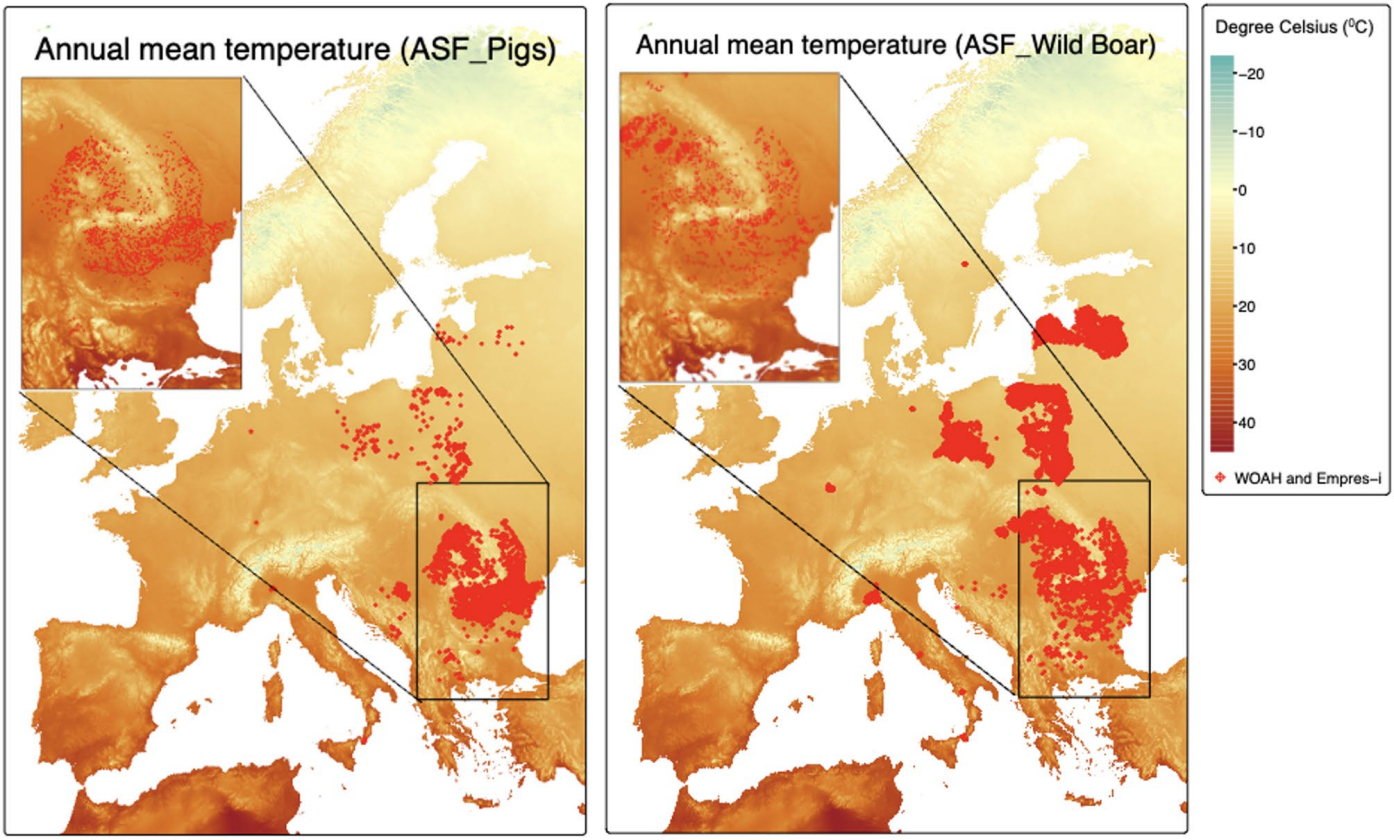
ASF cases (2018–2023) in pigs and boars overlaid on 2020 annual mean temperature map (°C)

In total, 5,626 ASF cases in domestic pigs were found in 11 European countries, compared to 19,395 cases reported in wild boar in 14 countries from 2018 to 2023 after combining the two datasets from WOAH-WAHIS and EMPRES-i. Most of the reported ASF cases in domestic pigs were in Romania (*n* = 3,831), followed by Bosnia and Herzegovina (*n* = 1,145) and Poland (*n* = 367), while the majority of ASF cases in wild boar were found in Poland (*n* = 9,699) and Romania (*n* = 2,229) (Table 1; Figs. 1, 2, 3 and 4). No strong correlation between the predictors (Additional files 3 and 4), and no multicollinearity was identified in the model (VIFs < 1). Spatial autocorrelation in the residuals was close to zero with values of 1.218 × 10⁻⁴ (P-value = 3.2 × 10⁻²) for domestic pigs and 1.214 × 10⁻² (P-value = 1.279 × 10⁻⁴) for wild boars. This indicates that there is no notable spatial autocorrelation (i.e., values are randomly distributed) for both WOAH-WAHIS and EMPRES-i, suggesting spatial patterns were well captured.

Results of the univariable analyses indicated a significant statistical association between ASF cases in wild boar and land landcovers (for details, Tables 2 and 3), while ASF in domestic pigs showed an association with temperature, pig density, human density, and landcover (for details, Tables 4 and 5). The correlation values between the predictors can be found in the supplement materials (Additional files 3 and 4).

**Table 3:** Univariable negative binomial model of (a)biotic factors influencing ASF in domestic pigs, 5 × 5 km

Species	Fixed effects	Estimate	Std. Error	Z-value	*P*-value	Odd ratio	Lower Conf.	Upper Conf.
Number of observations = 549, AIC= 3401.0, BIC = 3426.9, logLik= − 1694.5, deviance = 3389.0, df.resid = 543								
Pigs	Intercept	− 5.466	4.937	− 1.107	0.268	0.004	0.001	67.380
	Precipitation	2.485	1.740	1.428	0.153	11.996	0.396	362.946
	Random effects	Variance	Std.Dev.					
	Country (Intercept)	0.842	0.918					
	Longitude (Intercept)	0.211	0.459					
	Latitude (Intercept)	0.893	0.945					
Number of observations = 549, AIC = 3428.8, BIC = 38254.1, logLik= − 1695.5, deviance = 3391.0, df.resid = 543								
Pigs	Intercept	2.360	2.543	0.928	0.353	10.595	0.072	1548.701
	Temperature	− 0.611	1.940	− 0.315	0.753	0.543	0.012	24.314
	Random effects	Variance	Std.Dev.					
	Country (Intercept)	0.892	0.945					
	Longitude (Intercept)	0.208	0.456					
	Latitude (Intercept)	0.900	0.949					
Number of observations = 5585, AIC = 3403.0, BIC = 3428.8, logLik= − 1695.5, deviance = 3391.0, df.resid = 543								
Pigs	Intercept	1.766	0.698	2.529	0.011*	5.847	1.488	22.974
	Pig density	-0.066	0.199	− 0.330	0.741	0.936	0.634	1.383
	Random effects	Variance	Std.Dev.					
	Country (Intercept)	0.857	0.926					
	Longitude (Intercept)	0.206	0.454					
	Latitude (Intercept)	0.901	0.949					
Number of observations = 549, AIC = 3388.5, BIC = 3414.3, logLik= − 1688.2, deviance = 3376.5, df.resid = 543								
Pigs	Intercept	2.279	0.393	5.806	6.4e-09 ***	9.769	4.526	21.086
	Human density	− 0.458	0.119	− 3.844	0.00012 ***	2.442	6.264	43.205
	Random effects	Variance	Std.Dev.					
	Country (Intercept)	0.797	0.893					
	Longitude (Intercept)	1.056	1.028					
	Latitude (Intercept)	8.860e-08	0.0002					
Number of observations = 549, AIC = 3406.3, BIC = 3470.9, logLik= − 1688.2, deviance= 3376.3, df.resid = 534								
Pigs	Intercept	1.358	0.3742	3.630	0.0002***	3.890	1.868	8.098
	Industrial, commercial and transport units	− 0.812	0.706	− 1.150	0.250	0.444	0.111	1.772
	Mine, dump and construction sites	-0.759	0.508	-1.494	0.135	0.468	0.173	1.267
	Arable land	0.286	0.169	1.686	0.091.	1.331	0.955	1.855
	Permanent crops	− 0.173	0.334	− 0.519	0.604	0.841	0.437	1.617
	Pastures	0.511	0.317	1.614	0.107	1.667	0.896	3.100
	Heterogeneous agricultural areas	0.139	0.171	0.813	0.416	1.149	0.822	1.605
	Forests	0.450	0.197	2.281	0.022*	1.570	1.066	2.312
	Scrub and/or herbaceous vegetation associations	0.377	0.525	0.718	0.472	1.458	0.521	4.082
	Inland wetlands	− 0.460	0.789	− 0.582	0.560	0.631	0.134	2.967
	Inland waters	0.656	1.266	0.518	0.604	1.927	0.161	23.038
	Random effects	Variance	Std.Dev.					
	Country (Intercept)	0.846	0.919					
	Longitude (Intercept)	0.213	0.461					
	Latitude (Intercept)	0.834	0.913					

Signif. codes: 0 ‘***’ 0.001 ‘**’ 0.01 ‘*’ 0.05 ‘.’ 0.1 ‘ ’ 1

**Table 4:** Multivariable binomial logistic models of ASF occurrence in pigs and wild boar, 10 × 10 km

Species	Fixed effects	Estimate	Std. Error	Z-value	*P*-value	Odds Ratio	Lower Conf.	Upper Conf.
Number of observations = 552, AIC = 37828.2, BIC = 37954.1, logLik= − 18895.1, deviance = 37790.2, df.resid = 515								
Pigs	Intercept	− 1.106	0.656	− 1.688	0.091.	0.331	0.092	1.195
	Pig density	0.215	0.067	3.212	0.001**	1.087	1.087	1.414
	Temperature	1.611	0.549	2.931	0.003**	5.008	1.705	14.709
	Human density	− 0.715	0.162	− 4.418	9.94e-06***	0.489	0.356	0.672
	Industrial, commercial, and transport units	0.975	0.161	6.053	1.42e-09***	1.933	1.923	3.635
	Mine, dump, and construction sites	0.170	0.429	0.397	0.691	1.186	0.512	2.746
	Artificial, non-agricultural vegetated areas	− 0.049	0.689	− 0.071	0.943	0.952	0.246	3.678
	Arable land	0.580	0.058	10.071	< 2e-16***	1.787	1.596	1.787
	Permanent crops	− 0.024	0.127	− 0.188	0.850	1.787	1.596	2.001
	Pastures	0.252	0.097	2.582	0.009**	0.976	0.762	1.251
	Heterogeneous agricultural areas	0.191	0.069	2.738	0.006**	1.287	1.063	1.558
	Forests	0.330	0.108	3.051	0.002**	1.211	1.056	1.389
	Scrub and/or herbaceous vegetation associations	0.517	0.292	1.771	0.077.	1.391	1.125	1.720
	Inland wetlands	− 0.518	0.234	− 2.213	0.027*	1.677	0.946	2.973
	Inland waters	− 0.479	0.228	− 2.099	0.036*	0.596	0.376	0.942
	Random effects	Variance	Std.Dev.					
	Country (Intercept)	0.749	0.865					
	Longitude (Intercept)	1.014	1.007					
	Latitude (Intercept)	0.725	0.851					
Number of observations = 3636, AIC = 13962.2, BIC = 14073.8, logLik= − 6693.1, deviance = 13926.2, df.resid = 3618								
Wild boar	Intercept	0.453	0.197	2.297	0.022 *	1.573	1.069	2.316
	Industrial, commercial, and transport units	− 1.147	0.493	− 2.323	0.020*	0.318	0.121	0.836
	Mine, dump, and construction sites	0.126	0.358	0.353	0.724	1.135	0.562	2.289
	Artificial, non-agricultural vegetated areas	0.184	0.977	0.188	0.851	1.202	0.177	8.161
	Arable land	− 0.049	0.142	-0.344	0.731	0.952	0.720	1.259
	Permanent crops	0.113	0.217	0.519	0.603	1.119	0.732	1.712
	Pastures	− 0.137	0.157	− 0.865	0.387	0.873	0.642	1.187
	Heterogeneous agricultural areas	− 0.136	0.152	-0.895	0.371	0.873	0.648	1.175
	Forests	0.037	0.138	0.266	0.790	1.037	0.792	1.358
	Scrub and/or herbaceous vegetation associations	0.052	0.165	0.317	0.751	1.054	0.762	1.456
	Open spaces with little or no vegetation	0.226	0.649	0.348	0.727	1.254	0.351	4.474
	Inland wetland	1.342	0.409	3.278	0.001**	3.826	1.715	8.536
	Inland waters	0.252	0.270	0.934	0.350	1.287	0.758	2.184
	Marine waters	0.158	0.666	0.237	0.813	1.171	0.317	4.317
	Random effects	Variance	Std.Dev.					
	Country (intercept)	0.200	0.447					
	Longitude (Intercept)	0.122	0.349					
	Latitude (Intercept)	0.328	0.572					

Signif. codes: 0 ‘***’ 0.001 ‘**’ 0.01

N.B. Multivariable analysis included significant univariable predictors using ASF data from WOAH-WAHIS and EMPRES-i

**Table 5:** Multivariable binomial logistic models of ASF occurrence in pigs and wild boar, 5 × 5 km

Species	Fixed effects	Estimate	Std. Error	Z-value	*P*-value	Odds Ratio	Lower Conf.	Upper Conf.
Number of observations = 549, AIC = 3396.6, BIC = 3465.5, logLik= − 1682.3, deviance = 3364.6, df.resid = 533								
Pigs	Intercept	1.679	0.367	4.575	4.77e-06***	5.361	2.611	11.006
	Human density	− 1.590	0.462	− 3.440	0.0005***	0.204	0.082	0.505
	Industrial, commercial, and transport units	− 0.774	0.700	− 1.105	0.269	0.461	0.117	1.820
	Mine, dump, and construction sites	− 0.802	0.505	− 1.591	0.112	0.448	0.167	1.205
	Arable land	0.244	0.168	1.450	0.147	1.277	0.918	1.776
	Permanent crops	− 0.448	0.340	-1.316	0.188	0.639	0.328	1.245
	Pastures	0.291	0.320	0.908	0.364	1.338	0.714	2.506
	Heterogeneous agricultural areas	0.088	0.169	0.520	0.603	1.092	0.783	1.524
	Forests	0.266	0.203	1.312	0.189	1.305	0.877	1.944
	Scrub and/or herbaceous vegetation associations	0.115	0.525	0.219	0.827	1.122	0.400	3.144
	Inland wetlands	− 0.421	0.782	− 0.538	0.590	0.656	0.142	3.041
	Inland waters	0.692	1.253	0.552	0.581	1.998	0.171	23.301
	Random effects	Variance	Std.Dev.					
	Country (Intercept)	0.741	0.861					
	Longitude (Intercept)	0.204	0.452					
	Latitude (Intercept)	0.812	0.901					
Number of observations = 3636, AIC = 13962.2, BIC = 14073.8, logLik= − 663.1, deviance = 13926.2, df.resid = 3618								
Wild boar	Intercept	0.453	0.197	2.297	0.022 *	1.573	1.069	2.316
	Industrial, commercial, and transport units	− 1.147	0.493	− 2.323	0.020*	0.318	0.121	0.836
	Mine, dump, and construction sites	0.126	0.358	0.353	0.724	1.135	0.562	2.289
	Artificial, non-agricultural vegetated areas	0.184	0.977	0.188	0.851	1.202	0.177	8.161
	Arable land	− 0.049	0.142	− 0.344	0.731	0.952	0.720	1.259
	Permanent crops	0.113	0.217	0.519	0.603	1.119	0.732	1.712
	Pastures	− 0.137	0.157	− 0.865	0.387	0.873	0.642	1.187
	Heterogeneous agricultural areas	− 0.136	0.152	-0.895	0.371	0.873	0.648	1.175
	Forests	0.037	0.138	0.266	0.790	1.037	0.792	1.358
	Scrub and/or herbaceous vegetation associations	0.052	0.165	0.317	0.751	1.054	0.762	1.456
	Open spaces with little or no vegetation	0.226	0.649	0.348	0.727	1.254	0.351	4.474
	Inland wetland	1.342	0.409	3.278	0.001**	3.826	1.715	8.536
	Inland waters	0.252	0.270	0.934	0.350	1.287	0.758	2.184
	Marine waters	0.158	0.666	0.237	0.813	1.171	0.317	4.317
	Random effects	Variance	Std.Dev.					
	Country (intercept)	0.200	0.447					
	Longitude (Intercept)	0.122	0.349					
	Latitude (Intercept)	0.328	0.572					

Signif. codes: 0 ‘***’ 0.001 ‘**’ 0.01

N.B. Multivariable analysis included significant univariable predictors using ASF data from WOAH-WAHIS and EMPRES-i

Tables 4 and 5 show the results from the negative binomial multivariable analyses (estimated using ML and nlminb optimiser) to find an association between ASF cases and the potential drivers (Additional file 1). In total, four variables (pig density, temperature, human density, and the land cover types (‘industrial, commercial, and transport units’ and ‘inland wetlands’)) were significantly associated with ASF occurrence in domestic pigs. However, the type of landcover variable that is associated with the ASF occurrence differed between pig and wild boar populations.

The results of the negative binomial regression for the domestic pig reveal that a higher pig density (OR 1.087; CI 1.087–1.414), higher temperature (OR 5.008; Cl 0.1.705–14.709), and higher human density (OR 0.489; Cl 0.356–0.672) were associated with ASF cases. Land cover (industrial, commercial, and transport units (OR 1.933; CI 1.923–3.635), heterogeneous agricultural areas (OR 1.287; CI 1.063–1.558), arable land (OR 1.787; CI 1.596–1.787), and forests (OR 1.211; CI 1.056–1.389)) were significantly associated with ASF cases in domestic pigs (Table 3). No association was found between ASF occurrence in domestic pigs and the explanatory variable ‘participation’ (Table 3; Additional file 6). The mean accuracy of the model in the cross-validation was 88% (Additional file 5) for domestic pigs. For wild boar, the following land cover types (Table 4) were associated with ASF occurrence: industrial, commercial, and transport units (OR 0.318; CI 0.121–0.836) and inland wetlands (OR 3.826; CI 1.715–8.536)). The mean accuracy of the model in the cross-validation was 32% (Additional file 5) for wild boar.

When a finer spatial resolution (5 × 5 km) for all (a)biotic variables around the identified ASF cases was applied (i.e. Table 3 (univariate) and Table 5 (multivariate))—compared to the coarser resolution of 10 × 10 km (i.e. Table 2 (univariate) and Table 4 (multivariate)), the associations with (a)biotic variables for wild boars remained consistent. In contrast, for domestic pigs, only human population density remained significantly associated with ASF occurrence at this finer scale. The model accuracy for ASF in wild boars remained largely unchanged when using a finer spatial resolution (32%), whereas for domestic pigs, model accuracy decreased from 88% to 73%. In spatial risk models, even subtle spatial biases can mislead the identification of high-risk areas, resulting in misallocated resources or ineffective surveillance. Ensuring spatial accuracy is essential for reliable, targeted interventions and informed decision-making in disease control.

Figure 1 shows the spatial distribution of outbreaks reported between 2018 and 2023 in domestic pigs and wild boar populations in combination with pig and wild boar density (Fig. 1), human density (Fig. 2), landcover (Fig. 3), temperature (Fig. 4), and precipitation (Additional file 7). The spatial pattern of the residuals of the model predictions (Additional file 8). The shiny app that illustrates Figs. 1, 2, 3 and 4 in a more interactive way is available at: https://tipton-arpm.shinyapps.io/tipton-ASF/ [41].

## Discussion

WOAH-WAHIS and EMPRES-i provide extensive, global coverage of ASF occurrence data, allowing researchers to examine patterns on an international scale. By integrating these datasets with additional environmental and demographic information, regions at higher risk could be identified and prioritised for surveillance and intervention. Factors like domestic pig density, human density, temperature, and land cover further enhance the predictive accuracy of ASF occurrence. This, in turn, can support the development of early warning systems, improve risk assessments, and help optimise resource allocation. While dataflow designs, such as those presented in this study, offer advantages like integrating heterogeneous datasets to identify factors influencing disease occurrence, they also present challenges, including reporting biases and the merging of diverse datasets. While missing values for (a)biotic factors were imputed using the mean of neighbouring cells, the results are still influenced by reporting biases in the source databases. These stem from assumptions about unreported disease areas, which may include, e.g., regions where ASF exists but remains unreported. Such uncertainties emphasise the need for a cautious interpretation of the model’s spatial outputs in the present study.

Several studies used the public domain datasets by WOAH-WAHIS and EMPRES-i aimed at tracking global disease trends, including, e.g., avian influenza, foot-and-mouth disease, African swine fever [42–45], . The study used WOAH-WAHIS surveillance data and time-series models to forecast HPAI detections in European nations and demonstrated a seasonal change between 2021 and 2022 [46]. Using this database, the authors assessed the spatiotemporal distribution of HPAI outbreaks and identified high-risk regions in Europe. Another study demonstrated how publicly available datasets can be effectively used to assess the spatial and temporal risk of HPAIV transmission to Danish cattle via wild birds [47]. The development of disease-spread prediction models has significantly benefitted from WOAH-WAHIS and EMPRES-i. For example, a previous study combined these databases with trade and climatic data to forecast zoonotic and vector-borne disease outbreaks [48]. Their findings, which are in line with ours, indicate that climate data have impacted the spread of infectious diseases. Another study used both WOAH-WAHIS and EMPRES-i data to analyse the peste des petits ruminants and strategies for its eradication [42]. They proposed a uniform approach to data collection between Europe and Asia for future research. Furthermore, a study used WOAH-WAHIS and EMPRES-i data to investigate HPAI H5N1 and H5N8 in domestic and wild bird populations [49]. They added that extermination of domestic birds should remain a top priority to prevent future epizootics. Studies have utilised WOAH-WAHIS and EMPRES-i data to inform global and national disease control strategies [50]. Examining historical epidemic data might help policymakers better allocate resources for disease prevention and response [51–53]. For instance, to implement biosecurity measures and immunisation campaigns efficiently in different regions [54–56].

The analysis suggests that both anthropogenic and environmental factors significantly influence ASF occurrence. High pig population density and warmer temperatures may facilitate disease spread through increased contact rates or stress-induced susceptibility. Interestingly, the inverse relationship with human population density could reflect better biosecurity measures or more effective surveillance systems in densely populated areas, enabling earlier detection and response. ASF occurrence also appears linked to land use patterns, with land cover types such as pastures, arable land, and industrial or transportation zones serving as proxies for human and livestock activity. In contrast, the lower probability associated with inland wetlands may reflect limited pig farming in these areas. These can be attributed to several interconnected factors, such as that pig farming is often practiced extensively, fewer barriers exist to separate domestic pigs from wild boars, delayed detection and control, and transport and trade dynamics. These elements contribute to an environment where the risks of ASF transmission are increased even in areas with lower human and wild boar density, highlighting the significance of focused biosecurity measures and surveillance in these areas.

Using (a)biotic factors at a spatial resolution of 10 × 10 km around each recorded ASF case, the multivariate analysis indicated (Table 4) that most of the ASF cases in domestic pigs were associated in high-density pig areas (OR 1.087; CI 1.087–1.414) but were five times higher in regions with high temperatures (OR 5.008; CI 1.705–14.709) and 0.5 times higher in regions with high human densities (OR 0.489; CI 0.356–0.672). Seven land cover variables were found to be significantly associated with ASF in domestic pigs: arable land (OR 1.787; CI 1.596–1.787), industrial, commercial, and transportation units (OR 1.933; CI:1.923–3.635), heterogeneous agricultural areas (OR 1.287; CI 1.063–1.558), forest (OR 1.211; CI 1.056–1.389), inland wetlands (OR 1.677; CI 0.946–2.973), and finally inland waters (OR 0.596; 0.376–0.942). However, using the finer geographical solution of 5 × 5 km grid caused a significant association between ASF in domestic pigs and human density (OR 5.361; Cl 2.611–11.006).

There were only two land cover-related factors significantly associated with ASF in wild boar regardless of the geographical resolution. Using (a)biotic factors at a spatial resolution of 10 × 10 km around each recorded ASF case, the multivariate analysis indicated a statistically significant association with ASF occurrence in wild boars and “industrial, commercial, and transport units” regions with an OR of 0.318 (CI 0.121–0.836; Table 4)), which is consistent with the findings published in another study [57], and the land cover “inland wetlands” with 3.8 higher OR for ASF in wild boar (CI 1.715–8.536) and consistent with findings by others [25]. The consistent associations between (a)biotic variables and ASF occurrence in wild boars across both spatial resolutions suggest that (a)biotic drivers of ASF transmission in wild boars operate at broader spatial scales. This likely explains why changing the grid resolution from 10 × 10 km to 5 × 5 km does not significantly affect these associations. In contrast, domestic pigs are typically confined to farms or small holdings, making them more susceptible to fine-scale environmental influences. At a coarser resolution (10 × 10 km), broader regional variables—such as different land cover, pig density, human density and temperature—may appear associated due to spatial averaging. However, when analysed at a finer spatial resolution (5 × 5 km), this averaging effect is reduced, revealing that only highly localized factors—such as human population density, which may reflect human activity or farm-level management practices—remain statistically significant.

According to the European Food Safety Authority, the trend of ASF occurrence has localised spread on pasture in Romania [58]. Our results (10 × 10 km) reveal that forest and arable land had a positive impact on the spread of ASF in the EU. These findings are in line with studies conducted by others [25, 59]. The authors demonstrate that highly dense forest areas and woodlands contribute as risk factors for ASF outbreaks. The spread of ASF across forests, pastures, scrublands, and agricultural fields has increased in 26 countries in the EU, according to [60]. The study also enhanced an understanding of how these factors are related to the risks between domestic pigs and wild boar in intensive pig farming systems. Previous research has demonstrated an association between land cover and an increase in ASF outbreaks in certain EU countries [61–64]. These findings are in line with the present study, as this study demonstrates that specific land covers are an important factor for the ASF occurrence compared to other land covers [45, 50, 65].

In the present study, the model’s accuracy (88.05%) suggests that public domain datasets with 10 × 10 km resolution related to ASF occurrence in domestic pigs can be used as predictor variables, while for ASF in wild boar, the model accuracy was lower, with 32.02%. The high accuracy of the model for domestic pigs indicates that ASF outbreaks in pig farms are closely linked to the selected predictor variables, including human density. This can be attributed to the controlled nature of pig farming, where factors such as population density and human interaction directly influence disease transmission, making ASF easier to predict [65–69]. Additionally, ASF reporting in domestic pigs is likely more precise and systematic due to mandatory surveillance and stringent biosecurity measures in the veterinary public health sector. In contrast, the lower accuracy for ASF in wild boars suggests that other factors beyond the selected predictor variables contribute to disease spread in wild populations [70]. Wild boar populations are highly dynamic, with movement patterns shaped by food availability, migration, and interactions with infected carcasses—factors that may not be fully captured by the model. ASF transmission in wild boars is often driven by environmental contamination (e.g., contact with infected carcasses) rather than direct density-dependent spread, making it more challenging to predict. Moreover, ASF surveillance and reporting in wild boars may be inconsistent across regions, leading to potential biases in the dataset. To improve accuracy, the model for wild boars may need to incorporate additional ecological and behavioural factors, such as habitat connectivity, carcass persistence, consistent wild boar density data, and wild boar movement patterns. Enhancing the model with real-time tracking data, hunter surveillance reports, or genetic analysis of ASF virus strains could further refine model performance.

A limitation of this study is that the analysis was conducted separately for domestic and wild boars due to inconsistent wild boar density data across the EU. Several studies have reported that the spread of ASF in domestic pigs is only possible in the presence of wild boars [71–75]. Further, the limitation of this study was that the univariable model may also provide a poor fit to the WOAH-WAHIS and EMPRES-i datasets, which often have complex relationships that a single predictor cannot fully explain. These issues highlight the need to account for data complexity and confounding factors when modelling count data. To handle this problem, we proceeded to perform multivariable negative binomial regression while improving the data variability explanation and the spatial resolution of the data. Although publicly available databases are essential for risk assessments by veterinary public health authorities and for epidemiological studies in general, they do have certain limitations. Another study []highlight several factors that can impact data reliability, including inconsistent disease reporting, underreporting in certain countries, and variations in data completeness across regions [76]. These challenges may introduce biases or gaps in the research, potentially affecting the study’s results. To address these limitations and improve data accuracy, researchers often combine data from multiple sources to better understand disease outbreaks, transmission patterns, and associated risk factors [77]. However, this strategy can also introduce new sources of bias. Public datasets, while valuable for enabling large-scale, reproducible analyses of animal disease across time and regions due to their accessibility, often lack the granularity and completeness of private data [47, 65–79], which may limit the precision and reliability of epidemiological assessments. Notwithstanding their limitations, WOAH-WAHIS and EMPRES-i continue to be essential epidemiological data instruments, providing insightful information that helps early warning systems, guides policy choices, and promotes global health security.

## Conclusions

The model demonstrated high accuracy in analysing abiotic factors for ASF in pigs but performed poorly for wild boars, highlighting the limitations of publicly available data. Improving the data quality of the analysed factors, such as wild boar density, and incorporating restricted data on animal movements and carcass interactions could enhance predictions and improve control efforts. Assumptions about unreported disease areas such as regions with hosts but no reported cases or where ASF is present but undetected introduce uncertainties that necessitate cautious interpretation of the spatial outputs and underscore the need for more comprehensive, higher-quality data, including higher-resolution spatial inputs, to improve model accuracy. In this context, the fine geographic resolution of the 5 × 5 km grid compared to a coarser resolution of 10 × 10 km did not alter the study outcomes related to ASF in wild boars but reduced the number of associated factors linked to ASF in domestic pigs, suggesting that ASF in wild boars is influenced by broader-scale ecological factors, whereas in domestic pigs, transmission is more closely tied to localised conditions.

## Supplementary Information

Below is the link to the electronic supplementary material.

Supplementary Material 1.

## Data Availability

World Organisation for Animal Health (WOAH) (2024) – Periodical Data Extraction WAHIS SharePoint. Retrieved on 15-01-2024 from. https://oieoffice365.sharepoint.com/sites/PeriodicaldataextractionsOIE-WAHIS? e=1%3Aeb4624be3dd2463eb42952ef18f89504 Reproduced with permission. WOAH bears no responsibility for the integrity or accuracy of the data contained herein, but not limited to, any deletion, manipulation, or reformatting of data that may have occurred beyond its control. Data and model code of this study are available on a shiny app: https://tipton-arpm.shinyapps.io/tipton-ASF/.
