# Supplementary material for: Can public-domain datasets be leveraged to identify factors associated with the occurrence of African swine fever in europe?
Source: Acta Vet Scand. 2025 Nov 20;67:48. doi: 10.1186/s13028-025-00832-7 (PMC12632064; doi:10.1186/s13028-025-00832-7)
Supplement: Supplementary file 1 — Supplementary Material 1. [file 13028_2025_832_MOESM1_ESM.docx]

**Can public-domain datasets be leveraged to identify factors associated with the occurrence of African swine fever in Europe?**

**Ofosuhene Okofrobour Apenteng^1*^, Ana Rita Pinheiro Marques^1^, Lene Jung Kjær^1^, Beate Conrady^1^**

^1^Department of Veterinary and Animal Sciences, Section for Animal Welfare and Disease Control, University of Copenhagen, Denmark.

*Corresponding author

Ofosuhene Okofrobour Apenteng –ofap@sund.ku.dk; Ana Rita Pinheiro Marques – arpm@sund.ku.dk, Lene Jung Kjær—lenju@sund.ku.dk and Beate Conrady - bcon@sund.ku.dk.

Additional file1: Definition of the land cover variables for level 2.

| **Factors** | **Definition** [1] |
| --- | --- |
| Heterogeneous agricultural areas | These are areas of annual crops farmed under forest trees; regions of annual crops grown with permanent crops on the same piece of land. |
| Arable land | Lands under a rotation system used for annually harvested plants and fallow lands, which are rain-fed or irrigated. |
| Urban fabric | Is a place mostly inhabited by homes and structures used by public utilities or administration, as well as any areas related to them. |
| Pastures | Dense grass cover, of floral composition, dominated by graminaceae, not under a rotation system. |
| Inland wetlands | Standing water with a particular type of vegetation, such as low shrubs, semi-ligneous plants, or herbaceous plants. |
| Mine, dump and construction sites | This is an artificial area mostly used for extraction, building sites, artificial waste disposal sites, and the lands surrounding them. |
| Industrial, commercial and transport units | Is an area mainly occupied by industrial activities of manufacturing, transport infrastructures for road traffic and rail networks, and industrial pig density rearing facilities. |
| Permanent crops | Is every surface that is permanently planted with crops—not using a rotation method. |
| Forests | Is an ecosystem characterized by a dense community of trees. |
| Inland waters | Areas that are frequently flooded or at risk of flooding during the majority of the year by fresh, brackish, or standing water. |
| Scrub and/or herbaceous vegetation associations | Is a temperate shrubby area with Atlantic and alpine heaths, and shrubby formation with sparse trees. |
| Artificial, non-agricultural vegetated areas | Areas that people freely designate for leisure. includes athletic and recreational amenities, as well as green or recreational urban parks. |
| Marine waters | They are oceanic and continental shelf waters, bays and narrow channels including sea lochs, straits and estuaries. |

Additional file 2: Data used in the presented study are highlighted in gray.

| **WOAH** | **EMPRES-i** |
| --- | --- |
| epi_event_id | Event.ID |
| disease_id | Disease |
| reporting_level | Serotype |
| strain_eng | Region |
| strain_fr | Subregion |
| strain_esp | Country |
| sero_sub_genotype_eng | Admin.level.1 |
| sero_sub_genotype_fr | Locality |
| sero_sub_genotype_esp | Latitude |
| disease_eng | Longitude |
| disease_fr | Diagnosis.source |
| disease_esp | Diagnosis.status |
| iso_code | Animal.type |
| country | Species |
| region | Observation.date..dd.mm.yyyy. |
| reason of notification | Report.date..dd.mm.yyyy. |
| event_start date | Humans.affected |
| event_confirmation_date | Human.deaths |
| **event_closing_date** |  |
| date_last_occurrence |  |
| Terra_Aqua |  |
| Report_id |  |
| IN_FUR |  |
| Report_number |  |
| Report_Nat_Ref |  |
| Reporting_date |  |
| Outbreak_id |  |
| Outbreak_Nat_Ref |  |
| Outbreak_start_date |  |
| Outbreak_end_date |  |
| Is_cluster |  |
| N_outbreaks_cluster |  |
| Epi_unit |  |
| Longitude |  |
| Latitude |  |
| Location_aprox |  |
| Location_name |  |
| Ob_area_id |  |
| level3_area_id |  |
| level3_unique_code |  |
| level3_name |  |
| level2_area_id |  |
| level2_unique_code |  |
| level2_name |  |
| level1_area_id |  |
| **level1_unique_code** |  |
| level1_name |  |
| country_area_id |  |
| country_unique_code |  |
| country_name |  |
| is_wild |  |
| wild_type |  |
| water_type |  |
| Species |  |
| quantitative_unit |  |
| susceptible |  |
| cases |  |
| dead |  |
| killed_disposed |  |
| slaughtered |  |
| vaccinated |  |
| morbidity |  |
| mortality |  |

**
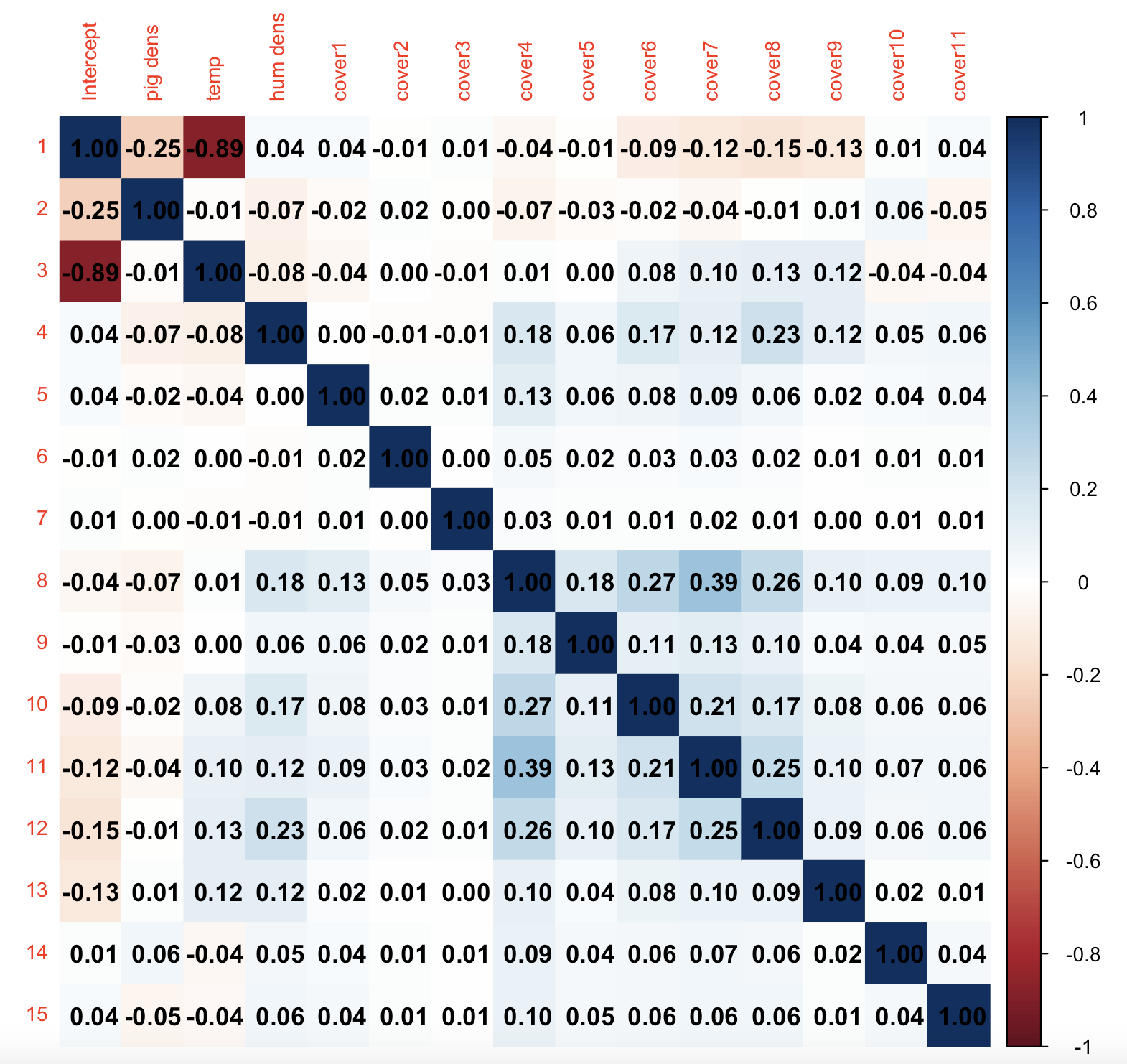
**

Additional file 3: Correlation between independent variables in the model for ASF in domestic pigs

Where pig dens=pig density, temp=temperature, hum dens=human density, cover1= Industrial/commercial and transport units, cover2= Mine/ dump and construction sites, cover3= Artificial/ non-agricultural vegetated areas, cover4= Arable land, cover5= Permanent crops, cover6= Pastures, cover7=Heterogeneous agricultural areas, cover8= Forests, cover9= Scrub and/or herbaceous vegetation associations, cover10= Inland wetlands, cover11= Inland waters.


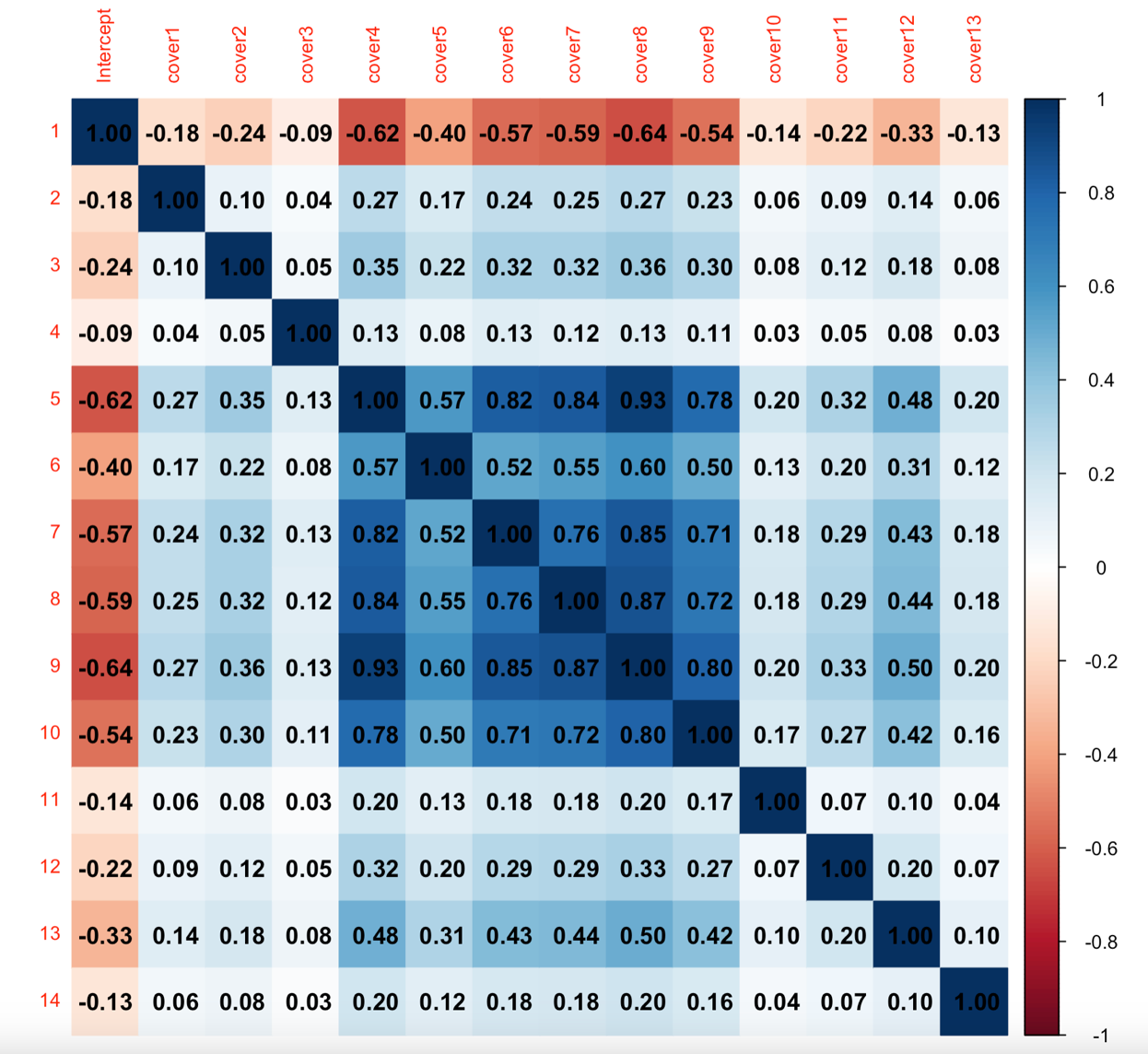


Additional file 4: Correlation between independent variables in the model for ASF in wild boar

Where cover1=Industrial/commercial and transport units, cover2=Mine/dump and construction sites, cover3=Artificial/non-agricultural vegetated areas, cover4=Arable land, cover5=Permanent crops, cover6=Pastures, cover7=Heterogeneous agricultural areas, cover8=Forests, cover9=Scrub and or herbaceous vegetation associations, cover10=Open paces with little or no vegetation, cover11=Inland wetlands, cover12=Inland waters, and cover13=Marine waters.

Additional file 5: Results for cross validation and confusion matrix for both domestic pigs (left) and wild boar (right)

|  | Domestic Pigs | | Wild Boar | |
| --- | --- | --- | --- | --- |
|  | Cross Validation | Confusion Matrix | Cross Validation | Confusion Matrix |
| Accuracy | 88.55% | 88.05% | 32.23% | 32.02% |
| Sensitivity | 99.89% | 99.99% | 99.99% | 99.99% |
| Specificity | 77.20% | 76.11% | 6.63% | 7.23% |
| Positive Prediction | 80.16% | 79.41% | 28.52% | 28.16% |
| Negative Prediction | 99.87% | 99.99% | 99.99% | 99.99% |
| Prevalence | 47.96% | 47.95% | 27.14% | 26.72% |
| Detection Rate | 47.91% | 47.95% | 27.14% | 26.72% |
| Detection Prevalence | 59.77% | 60.38% | 95.16% | 94.69% |

| 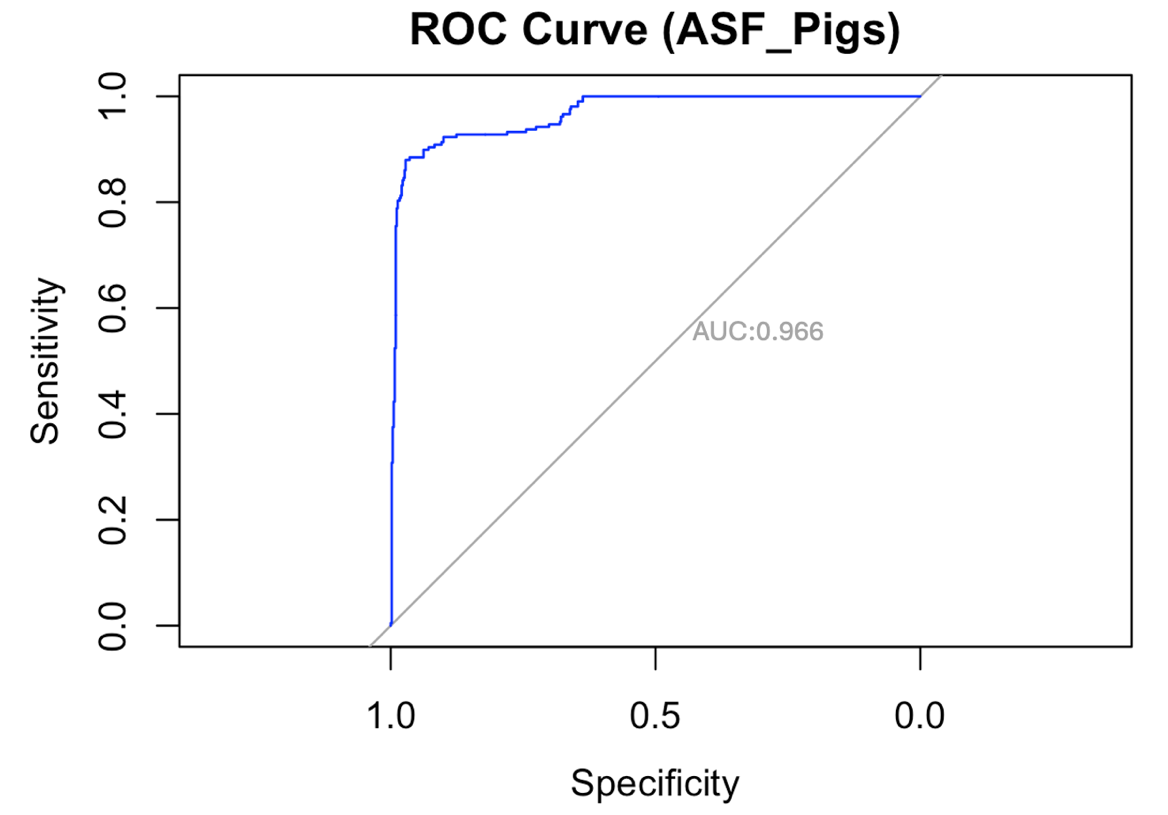 | 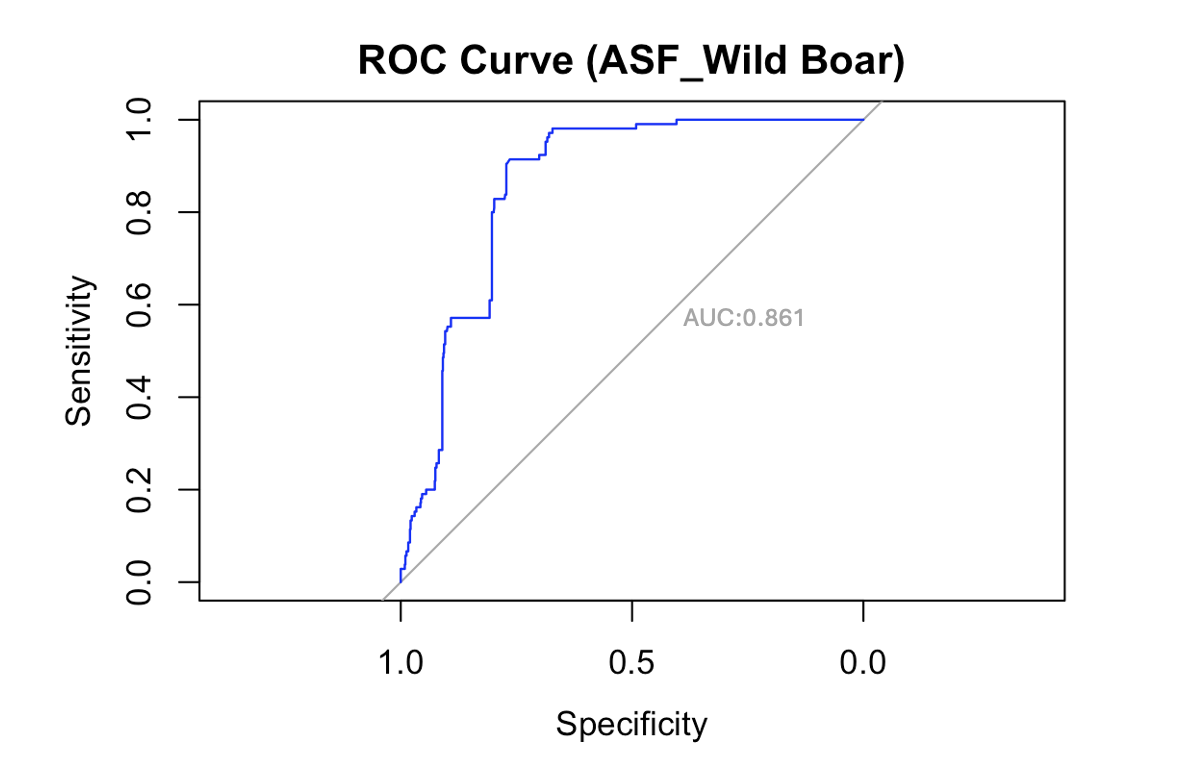 |
| --- | --- |

Additional file 6: Receiver Operating Characteristics (ROC) graph of the final mixed binomial logistic models for domestic pigs (left) and wild boar (right). Where AUC= Area under the curve.

The spatial distribution of outbreaks reported between 2018 and 2023 in domestic pigs and wild boar.


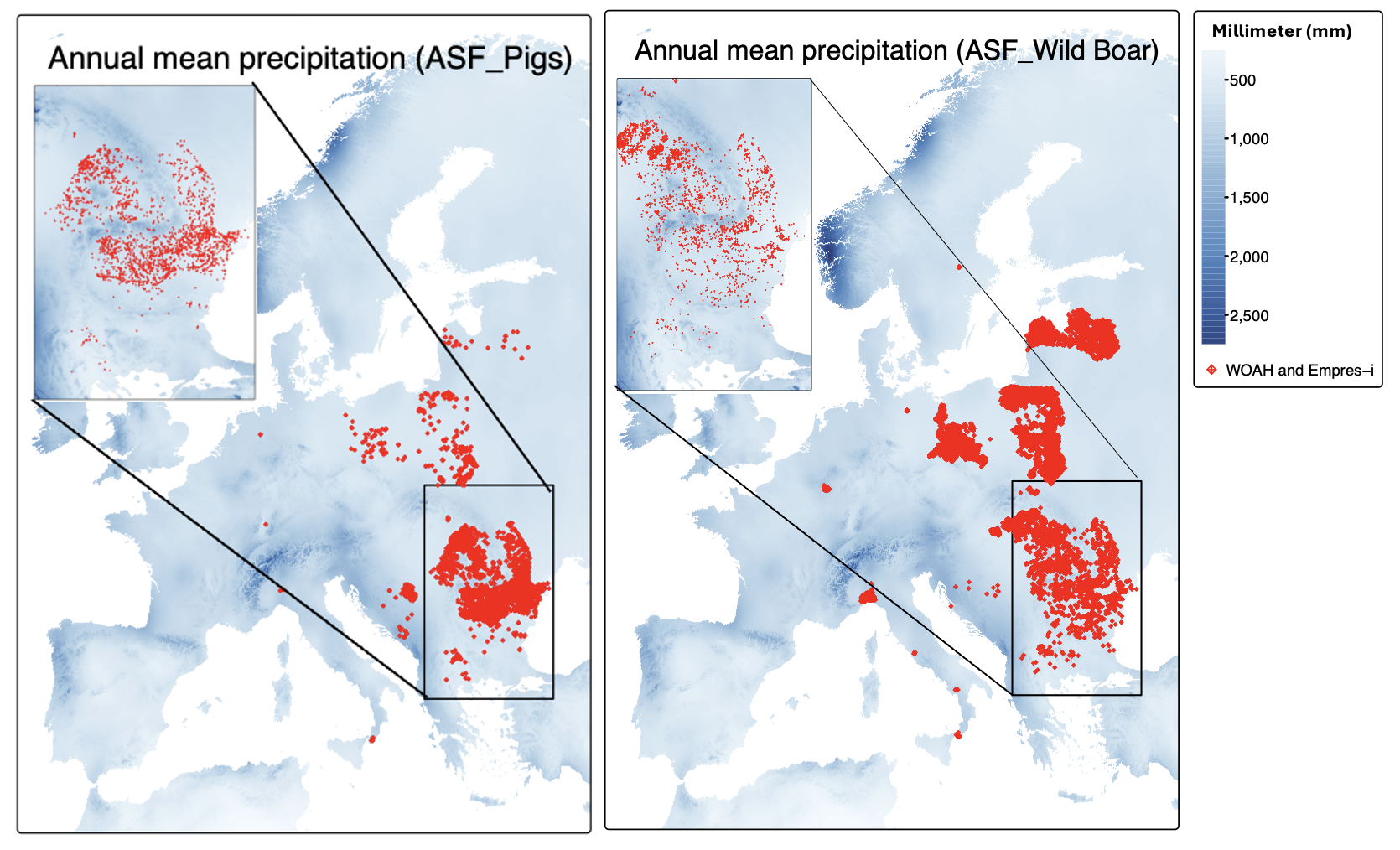


Additional file 7: Map of ASF-reported cases between 2018 and 2023 in domestic pigs (left) and wild boars (right) overlaid on the annual mean precipitation (millimetres (mm) in 2020)

The geographic spread of reported cases in wild boar, illustrates the spatial pattern of the residuals in the predictions.

Additional file 8: Map of ASF-reported cases of spatial pattern of the residuals in the predictions based on the residuals shown in background in the shaded area in EU of domestic pigs (left) and wild boar population (right).

Reference

1. Büttner G, Feranec J, Jaffrain G, Mari L, Maucha G, Soukup T. The Corine Land Cover 2000 Project. EARSeL eProceedings. 2004;3.
